# α-Tocopherol phosphate as a photosensitizer in the reaction of nucleosides with UV light: formation of 5,6-dihydrothymidine

**DOI:** 10.1186/s41021-022-00237-2

**Published:** 2022-02-15

**Authors:** Toshinori Suzuki, Chiaki Ono

**Affiliations:** School of Pharmacy, Shujitsu University, 1-6-1 Nishigawara, Okayama, 703-8516 Japan

**Keywords:** Tocopherol phosphate, Thymidine, Photosensitizer, UV light, Dihydrothymidine

## Abstract

**Introduction:**

α-Tocopherol phosphate, a natural water-soluble α-tocopherol analog, exists in biological tissues and fluids. Synthesized α-tocopherol phosphate is used as an ingredient of cosmetics.

**Findings:**

When a neutral mixed solution of 2′-deoxycytidine, 2′-deoxyguanosine, thymidine, and 2′-deoxyadenosine was irradiated with UV light at wavelengths longer than 300 nm in the presence of α-tocopherol phosphate, thymidine was markedly consumed in an α-tocopherol phosphate dose-dependent manner, whereas other nucleosides only slightly decreased. Two major product peaks were detected in an HPLC chromatogram. The products were identified as diastereomers of 5,6-dihydrothymidine. The addition of radical scavengers had almost no effects on the generation of 5,6-dihydrothymidine, whereas the reactions of nucleosides other than thymidine were suppressed. Trolox, another water-soluble α-tocopherol analog, did not generate 5,6-dihydrothymidine, although all nucleosides were slightly consumed. When UV irradiation of thymidine with α-tocopherol phosphate was conducted in D_2_O, two deuterium atoms were added to 5 and 6 positions of thymidine with both syn and anti configurations. The ratio of syn and anti configurations alternated depending on pD of the solution.

**Conclusions:**

The results indicate that α-tocopherol phosphate is a photosensitizer of nucleosides, especially thymidine, and that it introduces two hydrogen atoms to thymidine from H_2_O, generating 5,6-dihydrothymidine.

## Introduction

**Fig. 1:**
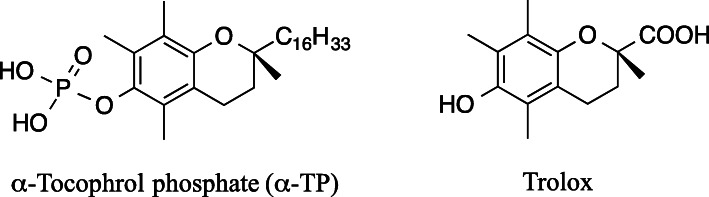
Structures of α-tocopherol phosphate (α-TP) and Trolox

α-Tocopherol phosphate (α-TP), also termed α-tocopheryl phosphate, is a water-soluble α-tocopherol analog, containing a hydroxy group of the chroman ring esterified by phosphoric acid. It is a naturally occurring chemical in animals and plants [1]. α-TP was detected in liver and adipose tissues of the rat [2]. It was also detected in human plasma, and the concentration markedly increased with α-TP supplementation [3]. Protective effects of α-TP against cell damage caused by peroxidants and UV irradiation were reported [4–6]. Synthesized α-TP is used in cosmetics due to its antioxidant and skin-conditioning activities [7]. The major risk factor for skin cancer in humans is prolonged exposure to UV light from the sun [8, 9]. DNA directly absorbs UV light at wavelengths shorter than 300 nm to generate photoproducts [10, 11]. However, due to almost complete absorption by the atmosphere, the strength of UV light at wavelengths shorter than 300 nm in sunlight is very low on the ground surface [8]. This suggests that substances causing photosensitization, photosensitizers, are involved in the mechanism of skin cancer. We recently reported that salicylic acid and uric acid are photosensitizers of the reaction of nucleosides with UV light at wavelengths longer than 300 nm [12, 13]. For salicylic acid, thymidine (dThd) resulted in cyclobutane dThd dimers *via* energy transfer. For uric acid, 2′-deoxycytidine (dCyd) resulted in products including a cyclic-amide adduct of dCyd *via* the formation of a radical from uric acid. In the present study, we show that α-TP is a photosensitzer on the reaction of nucleosides by UV irradiation at wavelengths longer than 300 nm and that the major products from dThd are diastereomers of 5,6-dihydrothymidine (DHdThd). As a control compound, we used Trolox, another water-soluble α-tocopherol analog, substituting a hydrocarbon chain (C_16_H_33_) for a carboxy group. It has been reported that Trolox acts as a multiple free radical scavenger [14]. Under the present experimental conditions, Trolox did not generate DHdThd by UV irradiation. The structures of α-TP and Trolox are shown in Fig. 1.

## Materials and methods

### Materials

dCyd, 2′-deoxyguanosine (dGuo), dThd, 2′-deoxyadenosine (dAdo), (±)-α-tocopherol phosphate disodium, Trolox ((±)-6-hydroxy-2,5,7,8-tetramethylchromane-2-carboxylic acid), and D_2_O (99.9 atom % D) were purchased from Sigma-Aldrich (MO, USA). Other chemicals were obtained from Sigma-Aldrich, Nacalai Tesque (Kyoto, Japan), and Tokyo Chemical Industry (Tokyo, Japan). Water was purified with a Millipore Milli-Q deionizer (MA, USA).

### Irradiation conditions

UV light generated by a high-pressure mercury lamp (250 W, SP9-250UB, Ushio, Tokyo, Japan) with an optical filter through a light guide was used to directly irradiate the surface of a solution (1 mL) in a glass vial (12 mm i.d.) at 37°C. The longpass optical filter had a cut-off of 300 nm (LU0300, Asahi Spectra, Tokyo, Japan) was used. The intensity of radiation on the surface of the sample solution was measured with a photometer (UIT-150, Ushio, Tokyo, Japan) equipped with a sensor UVD-S254 or UVD-S365. The intensities of UV light were 2 mW/cm^2^ for 254 nm and 408 mW/cm^2^ for 365 nm.

### HPLC, MS, and NMR conditions

An HPLC system including an SPD-M10Avp UV/Vis photodiode-array detector (Shimadzu, Kyoto, Japan) with an Inertsil ODS-3 octadecylsilane column of size 4.6 × 250 mm and particle size of 5 μm (GL Sciences, Tokyo, Japan) was used. The eluent was 20 mM ammonium acetate (pH 7.0) containing methanol. The methanol concentration was increased from 0 to 50% over 30 min in linear gradient mode. The column temperature was 40°C and the flow rate was 1 mL/min. The RP-HPLC chromatogram was detected at 200–500 nm. ESI-TOF-MS and HR-ESI-TOF-MS measurements were performed on a MicrOTOF spectrometer (Bruker, Bremen, Germany) in negative mode. The sample isolated by RP-HPLC was directly infused into the MS system by a syringe pump without a column. The NMR spectra were obtained on a JNM-ECA500 spectrometer (JEOL, Tokyo, Japan).

### pH and pD values

pH values were measured by a pH meter (F-22, Horiba, Tokyo, Japan) with a glass electrode (9618S-10D, Horiba). pD values of D_2_O solution were determined by adding 0.41 to the value on the pH meter [15].

#### Spectrometric data

Product **1** ((5*S*)-5,6-dihydrothymidine, 5*S*-DHdThd). ESI-TOF-MS (negative mode): m/z 243. HR-ESI-TOF-MS (negative mode): m/z 243.098607 obsd, (calcd for C_10_H_15_N_2_O_5_^–^ 243.098645). UV: *λ*_max_ = 214 nm (pH 7.0). ^1^H NMR (500 MHz, D_2_O): *δ* (ppm/TMSP-*d*_4_) 6.28 (dd, *J* = 7.2, 7.2 Hz, 1H, H-1′), 4.37 (m, 1H, H-3′), 3.91 (m, 1H, H-4′), 3.75 (m, 1H, H-5′ or 5′′), 3.68 (m, 1H, H-5′ or 5′′), 3.57 (dd, *J* = 5.7, 12.6 Hz, 1H, H-6a), 3.26 (dd, *J* = 9.5, 12.9 Hz, 1H, H-6b), 2.84 (m, 1H, H-5), 2.33 (m, 1H, H-2′ or 2′′), 2.15 (m, 1H, H-2′ or 2′′), 1.21 (d, *J* = 7.5 Hz, 3H, CH_3_). ^13^C NMR (125 MHz, D_2_O): *δ* (ppm/ TMSP-*d*_4_) 179.7 (C-4), 157.2 (C-2), 88.0 (C-4′), 86.7 (C-1′), 73.7 (C-3′), 64.5 (C-5′), 44.8 (C-6), 38.2 (C-2′), 37.5 (C-5), 15.0 (CH_3_).

Product **2** ((5*R*)-5,6-dihydrothymidine, 5*R*-DHdThd). ESI-TOF-MS (negative mode): m/z 243. HR-ESI-TOF-MS (negative mode): m/z 243.100093 obsd, (calcd for C_10_H_15_N_2_O_5_^–^ 243.098645). UV: *λ*_max_ = 214 nm (pH 7.0). ^1^H NMR (500 MHz, D_2_O): *δ* (ppm/TMSP-*d*_4_) 6.26 (dd, *J* = 7.2, 7.2 Hz, 1H, H-1′), 4.37 (m, 1H, H-3′), 3.91 (m, 1H, H-4′), 3.77 (m, 1H, H-5′ or 5′′), 3.69 (m, 1H, H-5′ or 5′′), 3.61 (dd, *J* = 6.3, 12.6 Hz, 1H, H-6b), 3.24 (dd, *J* = 10.9, 12.1 Hz, 1H, H-6a), 2.84 (m, 1H, H-5), 2.28 (m, 1H, H-2′ or 2′′), 2.12 (m, 1H, H-2′ or 2′′), 1.21 (d, *J* = 6.6 Hz, 3H, CH_3_). ^13^C NMR (125 MHz, D_2_O): *δ* (ppm/TMSP-*d*_4_) 179.4 (C-4), 157.0 (C-2), 88.0 (C-4′), 86.8 (C-1′), 73.7 (C-3′), 64.4 (C-5′), 44.8 (C-6), 38.0 (C-2′), 37.4 (C-5), 14.4 (CH_3_).

Product **1**′ ((5*S*)-5,6-dideuteriothymidine, 5*S*-DDdThd). ESI-TOF-MS (negative mode): m/z 245. HR-ESI-TOF-MS (negative mode): m/z 245.110927 obsd, (calcd for C_10_H_13_D_2_N_2_O_5_^–^ 245.111199). UV: *λ*_max_ = 215 nm (pH 7.0). ^1^H NMR (500 MHz, D_2_O): *δ* (ppm/TMSP-*d*_4_) 6.28 (dd, *J* = 7.2, 7.2 Hz, 1H, H-1′), 4.37 (m, 1H, H-3′), 3.90 (m, 1H, H-4′), 3.76 (m, 1H, H-5′ or 5′′), 3.68 (m, 1H, H-5′ or 5′′), 3.55 (s, 0.37H, H-6a), 3.24 (s, 0.63H, H-6b), 2.33 (m, 1H, H-2′ or 2′′), 2.15 (m, 1H, H-2′ or 2′′), 1.21 (s, 3H, CH_3_).

Product **2**′ ((5*R*)-5,6-dideuteriothymidine, 5*R*-DDdThd). ESI-TOF-MS (negative mode): m/z 245. HR-ESI-TOF-MS (negative mode): m/z 245.111205 obsd, (calcd for C_10_H_13_D_2_N_2_O_5_^–^ 245.111199). UV: *λ*_max_ = 215 nm (pH 7.0). ^1^H NMR (500 MHz, D_2_O): *δ* (ppm/TMSP-*d*_4_) 6.26 (dd, *J* = 7.2, 7.2 Hz, 1H, H-1′), 4.38 (m, 1H, H-3′), 3.91 (m, 1H, H-4′), 3.77 (m, 1H, H-5′ or 5′′), 3.69 (m, 1H, H-5′ or 5′′), 3.59 (s, 0.64H, H-6b), 3.23 (s, 0.36H, H-6a), 2.29 (m, 1H, H-2′ or 2′′), 2.12 (m, 1H, H-2′ or 2′′), 1.20 (s, 3H, CH_3_).

### Quantitative procedures

For the reactions of nucleoside mixtures, the concentrations of nucleosides were evaluated according to the integrated peak areas on RP-HPLC chromatograms detected at 260 nm compared with the peak areas of the starting nucleoside mixture. The concentrations of the products were evaluated according to the integrated peak areas on RP-HPLC chromatograms detected at 230 nm and by the molecular extinction coefficients at 230 nm (*ε*_230 nm_) compared with those of dThd. The reported *ε*_230 nm_ values of 1500 M^–1^ cm^–1^ for 5*S*-DHdThd, 1460 M^–1^ cm^–1^ for 5*R*-DHdThd, and 2650 M^–1^ cm^–1^ for dThd were used [16].

## Results and Discussion

**Fig. 2:**
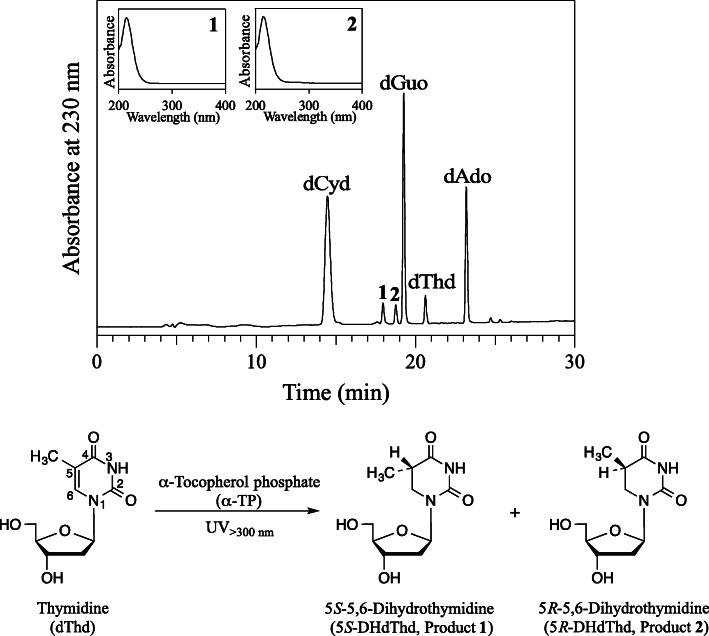
RP-HPLC chromatogram of a reaction mixture of nucleosides with α-TP detected at 230 nm (upper figure) and the reaction scheme (lower figure). A solution of 100 μM each of dCyd, dGuo, dThd, dAdo, and 5 mM α-TP was irradiated with UV through a 300-nm longpass filter in 100 mM potassium phosphate buffer at pH 7.4 and 37°C for 10 min. The insets are the UV spectra of Products **1** and **2**. Reaction of dThd irradiated by UV in the presence of α-TP

**Fig. 3:**
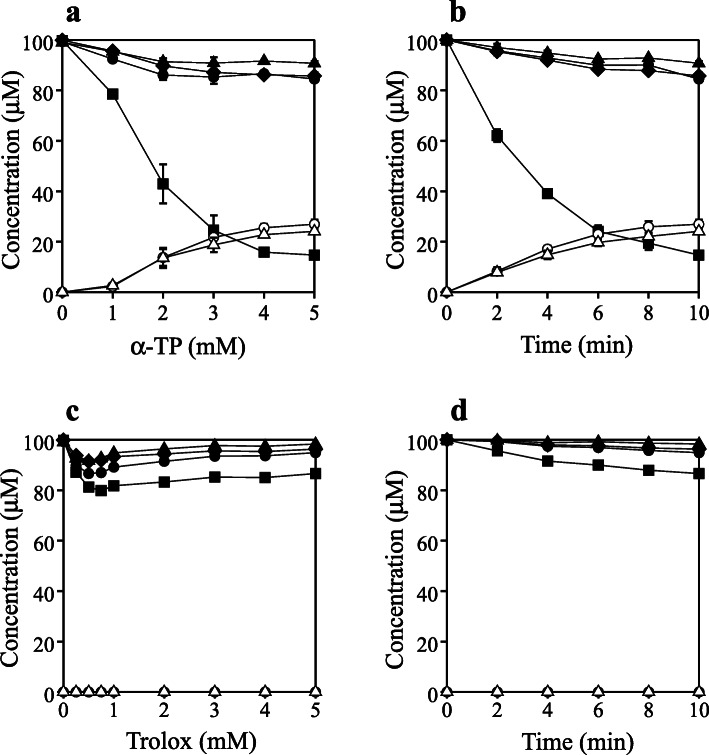
(**a**) α-TP dose-dependence of concentration changes in nucleosides and products when a solution of 100 μM each of dCyd, dGuo, dThd, and dAdo with 0–5 mM α-TP was irradiated with UV light through a 300-nm longpass filter for 10 min at pH 7.4 and 37°C. (**b**) Time-course of the concentration changes in nucleosides when a solution of 100 μM each of dCyd, dGuo, dThd, and dAdo with 5 mM α-TP in 100 mM potassium phosphate buffer at pH 7.4 was irradiated with UV light through a 300-nm longpass filter for 0–10 min at a temperature of 37°C. (**c**) Trolox dose-dependence of the concentration changes in nucleosides and products when a solution of 100 μM each of dCyd, dGuo, dThd, and dAdo with 0–5 mM Trolox was irradiated with UV light through a 300-nm longpass filter for 10 min at pH 7.4 and 37°C. (**d**) Time-course of the concentration changes in nucleosides when a solution of 100 μM each of dCyd, dGuo, dThd, and dAdo with 5 mM Trolox in 100 mM potassium phosphate buffer at pH 7.4 was irradiated with UV light through a 300-nm longpass filter for 0–10 min at a temperature of 37°C. dCyd (closed circles), dGuo (closed triangles), dThd (closed squares), dAdo (closed rhombi), 5*S*-DHdThd (Product **1**) (open circles), and 5*R*-DHdThd (Product **2**) (open triangles). All the reaction mixtures were analyzed by RP-HPLC. Means ± standard deviation (S.D.) (*n* = 3) are presented

dCyd, dGuo, dThd, and dAdo (100 μM each) with 5 mM α-TP in 100 mM potassium phosphate buffer at pH 7.4 and 37°C were irradiated with UV light from a Hg lamp through a 300-nm longpass filter for 10 min. Figure 2 shows the reversed phase (RP)-HPLC chromatogram of the reaction mixture detected at 230 nm. The concentration of dThd markedly reduced, while other nucleosides slightly decreased. Two product peaks with retention times of 17.9 and 18.7 min, referred to as Products **1** and **2**, respectively, were detected. UV spectra of the products are shown in the Fig. 2 insets. The products were isolated by RP-HPLC and subjected to MS and NMR. For both Products **1** and **2**, electrospray ionization time of flight mass spectrometry (ESI-TOF-MS) showed a signal at m/z 243 in negative mode. The high-resolution (HR)-ESI-TOF-MS values of the molecular ion agreed with the theoretical molecular mass for C_10_H_15_N_2_O_5_^–^ attributable to [dThd + 2H − H^+^]^−^ within 3 ppm. ^1^H and ^13^C NMR spectra of Products **1** and **2** were similar to those reported for diastereomers of 5,6-dihydrothymidine (DHdThd) [16]. Based on the reported values of chemical shifts and coupling constants of two H6 signals, Products **1** and **2** were identified as (5*S*)-5,6-dihydrothymidine (5*S*-DHdThd) and (5*R*)-5,6-dihydrothymidine (5*R*-DHdThd), respectively [17]. α-TP dose and irradiation time dependences on the UV reaction of nucleosides were examined. Figure 3**a** shows the α-TP dose-dependent changes in concentrations of nucleosides and products. A solution of 100 μM each of dCyd, dGuo, dThd, and dAdo with 0–5 mM α-TP in 100 mM potassium phosphate buffer at pH 7.4 was irradiated with UV light through a 300-nm longpass filter at a temperature of 37°C for 10 min. The concentrations were determined by RP-HPLC. With 0 mM α-TP, no reaction was observed. With an increasing α-TP concentration, the consumption of nucleosides increased. Especially, dThd decreased markedly. The products 5*S*-DHdThd and 5*R*-DHdThd, with similar amounts, increased with an increasing concentration of α-TP. Figure 3**b** shows the UV irradiation time dependence of nucleosides and product concentrations. A solution of 100 μM each of nucleosides with 5 mM α-TP was irradiated with UV light at pH 7.4 and 37°C for 0–10 min. With an increasing irradiation time, increased consumptions of nucleosides and yields of DHdThd were observed. These results indicate that α-TP is a photosensitzer on the reaction of nucleosides, especially dThd, by UV irradiation at wavelengths longer than 300 nm and that the major products from dThd are diastereomers of DHdThd. It has been reported that 5'-triphosphate of DHdThd is incorporated into DNA in place of 5'-triphosphate of dThd by Pol I Klenow fragment, although the rate is one order lower than that of 5'-triphosphate of dThd [18].Although single DHdThd in the DNA template is not a strong mutagenic or lethal lesion *in vivo*, multiple DHdThd in DNA should block DNA synthesis [19, 20].

As a control, similar experiments were conducted for Trolox. Figure 3**c** shows Trolox dose-dependent changes in concentrations of nucleosides and products, when a solution of 100 μM each of dCyd, dGuo, dThd, and dAdo with 0–5 mM Trolox in 100 mM potassium phosphate buffer at pH 7.4 and 37°C was irradiated with UV light for 10 min. With around 0.5 mM Trolox, maximal consumptions of nuleosides were observed. On increasing the Trolox concentration from 1 to 5 mM, the consumption of nucleosides was slightly suppressed. Diastereomers of DHdThd were not detected; the detection limit was 1 μM. Figure 3**d** shows the UV irradiation time-dependence of nucleosides and product concentrations. A solution of 100 μM of each nucleoside with 5 mM Trolox was irradiated with UV light at pH 7.4 and 37°C for 0–10 min. With an increasing irradiation time, the consumption of nucleosides increased, while no DHdThd was observed. The results suggest that the phosphate group on α-TP may make some contribution to the addition of hydrogen atoms to dThd, since Trolox, with no phosphate group in its structure, generated no DHdThd.

To obtain information on the reaction mechanism, UV irradiation reactions of nucleosides were conducted in the presence of ethanol (EtOH), a scavenger of radicals, and sodium azide (NaN_3_), a scavenger of radicals and singlet oxygen [21–25]. Table 1 shows the effects of EtOH and NaN_3_ on the UV irradiation reactions of nucleosides with α-TP. A mixed nucleoside (100 μM each) solution with 5 mM α-TP in 100 mM potassium phosphate buffer at pH 7.4 and 37°C with or without 1% EtOH and 10 mM NaN_3_ was irradiated with UV light for 10 min. Both EtOH and NaN_3_ suppressed the reactions of dCyd, dGuo, and dAdo, whereas little or no effects on the reaction of dThd were observed. Table 2 shows the effects of EtOH and NaN_3_ on the UV irradiation reactions of nucleosides with Trolox. The mixed nucleoside solution with 5 mM Trolox in 100 mM potassium phosphate buffer at pH 7.4 and 37°C with or without 1% EtOH and 10 mM NaN_3_ was irradiated with UV light for 10 min. Both EtOH and NaN_3_ suppressed the reactions of dCyd, dGuo, and dAdo, whereas few effects on the reaction of dThd were observed. The results suggest that the reactions of dCyd, dGuo, and dAdo by α-TP and Trolox are caused by radicals, whereas the reaction of dThd by α-TP resulting in DHdThd did not occur by radicals or singlet oxygen, suggesting that it did occur *via* energy transfer.

**Table 1:** Effects of additives on reactions of nucleosides and α-TP with UV light (>300 nm)^a^

	dCyd	dGuo	dThd	dAdo	5*S*-DHdThd	5*R*-DHdThd
Additives	(μM)	(μM)	(μM)	(μM)	(μM)	(μM)
None	84.5 ± 1.1	90.7 ± 0.9	14.7 ± 1.7	85.8 ± 1.4	27.0 ± 1.8	24.1 ± 1.2
1% EtOH	100.1 ± 0.4	98.6 ± 0.3	21.9 ± 3.5	91.8 ± 0.6	26.8 ± 1.9	24.0 ± 1.6
10 mM NaN_3_	100.1 ± 0.6	99.6 ± 0.5	24.9 ± 5.2	96.0 ± 0.8	24.3 ± 1.7	21.8 ± 1.3

^a^The mixed nucleoside solution with 5 mM α-TP in 100 mM potassium phosphate buffer at pH 7.4 and 37°C with or without 1% EtOH and 10 mM NaN_3_ was irradiated with UV light for 10 min. All reaction mixtures were analyzed by RP-HPLC. Means ± standard deviation (S.D.) (*n* = 3) are presented.

**Table 2:** Effects of additives on reactions of nucleosides and Trolox with UV light (>300 nm)^a^

	dCyd	dGuo	dThd	dAdo	5*S*-DHdThd	5*R*-DHdThd
Additives	(μM)	(μM)	(μM)	(μM)	(μM)	(μM)
None	94.9 ± 0.2	98.4 ± 0.2	86.6 ± 0.8	96.3 ± 0.3	<1	<1
1% EtOH	99.2 ± 0.4	100.2 ± 0.5	86.9 ± 0.6	98.1 ± 0.5	<1	<1
10 mM NaN_3_	100.4 ± 0.5	100.7 ± 0.5	92.8 ± 0.6	101.0 ± 0.4	<1	<1

^a^The mixed nucleoside solution with 5 mM Trolox in 100 mM potassium phosphate buffer at pH 7.4 and 37°C with or without 1% EtOH and 10 mM NaN_3_ was irradiated with UV light for 10 min. All reaction mixtures were analyzed by RP-HPLC. Means ± standard deviation (S.D.) (*n* = 3) are presented.

**Fig. 4:**
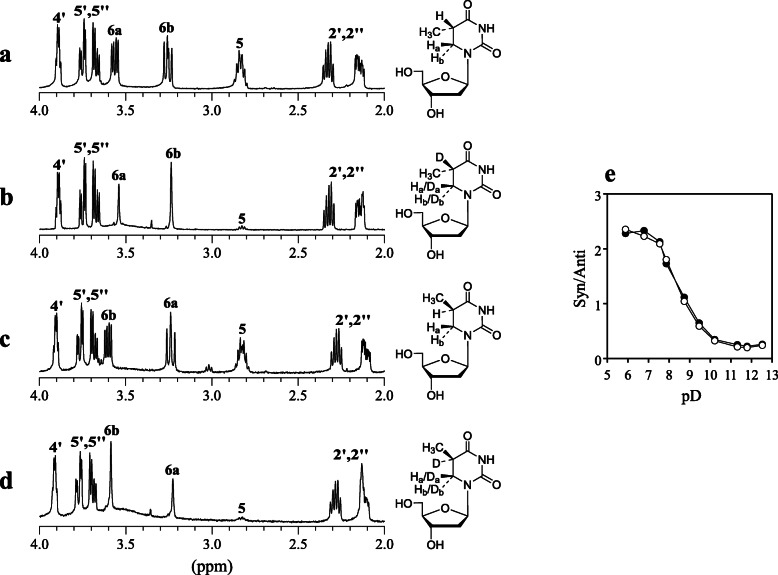
^1^H-NMR spectra ranging chemical shift of 2.0–4.0 ppm and structures of (**a**) 5*S*-DHdThd (Product **1**), (**b**) 5*S*-DDdThd (Product **1**′), (**c**) 5*R*-DHdThd (Product **2**), and (**d**) 5*R*-DDdThd (Product **2**′). (**e**) pD dependence of D_a_/D_b_ values on addition of a deuterium atom to the 6 position of dThd when a 99.9% D_2_O solution of 3 mM dThd, 7 mM α-TP, and 100 mM potassium phosphate buffer at pD 5.9–12.5 was irradiated with UV light at 37°C for 60 min. 5*S*-DDdThd (Product **1**′) (closed circles) and 5*R*-DDdThd (Product **2**′) (open circles). The D_a_/D_b_ values were calculated from integral values of H-6a and H-6b signals of ^1^H NMR

To obtain information about the origin and stereochemistry of hydrogen atoms added to dThd forming DHdThd, UV irradiation of dThd with α-TP was conducted in D_2_O. A 99.9% D_2_O solution of 3 mM dThd, 7 mM α-TP, and 100 mM potassium phosphate buffer at pD 7.9 was irradiated with UV light at 37°C for 60 min. Two product peaks (Products **1**′ and **2**′) with the same retention times as diastereomers of DHdThd (Products **1** and **2**) were isolated by RP-HPLC and subjected to MS and NMR analyses. For both Products **1**′ and **2**′, ESI-TOF-MS showed a signal at m/z 245 in negative mode. The HR-ESI-TOF-MS value of the molecular ion agreed with the theoretical molecular mass for C_10_H_13_D_2_N_2_O_5_^–^ attributable to [dThd + 2D − H^+^]^−^ within 3 ppm. Figure 4**a** and **b** shows ^1^H-NMR spectra of 2–4 ppm with their structures of Product **1** formed in H_2_O and Product **1**′ formed in D_2_O, respectively. For the base moiety of Product **1**, an H-5 multiplet signal and H-6a and H-6b double-doublet signals with intensity of one proton each were observed. For Product **1**′, the H-5 signal almost disappeared, although a weak signal attributable to H-5 was observed, probably due to residual H_2_O in the reaction solution. Singlet signals attributable to H-6a and H-6b with a total integral value of one proton were observed with a ratio of 0.37:0.63 (H-6a:H-6b). The result indicates that Product **1**′ is (5*S*)-5,6-dideuteriothymidine (5*S*-DDdThd), in which one deuterium atom was added to the 5-position of dThd and another deuterium atom was added to 6a- and 6b-positions with a ratio of 0.63:0.37 (D-6a:D-6b); therefore, the value of D-6a/D-6b (D_a_/D_b_) is 1.7. Figure 4**c** and **d** shows ^1^H NMR spectra of 2–4 ppm with their structures of Products **2** and **2**′, respectively. Similar to the case of Products **1** and **1**′, H-6a and H-6b double-doublet signals of Product **2** changed to singlet signals in Product **2**′ with a total integral value of one proton were observed with a ratio of 0.36:0.64 (H-6a:H-6b). The result indicates that Product **2**′ is (5*R*)-5,6-dideuteriothymidine (5*R*-DDdThd) in which one deuterium atom was added to the 5-position of dThd and another deuterium atom was added to 6a- and 6b-positions with a ratio of 0.64:0.36 (D-6a:D-6b); therefore, the value of D_a_/D_b_ is 1.8. These results suggest that, when the reaction occurred in H_2_O, two hydrogen atoms originating from H_2_O were added to dThd with both syn and anti configurations.

Then, pD-dependent changes in the value of D_a_/D_b_ were examined. A 99.9% D_2_O solution of 3 mM dThd, 7 mM α-TP, and 100 mM potassium phosphate buffer at pD 5.9–12.5 was irradiated with UV light at 37°C for 60 min. Generated 5*S*-DDdThd and 5*R*-DDdThd were isolated by RP-HPLC and subjected to NMR analysis. Figure 4**e** shows the pD-dependent changes in the value of D_a_/D_b_ for deuterium addition calculated from integral values of H-6a and H-6b signals. Under neutral and mildly acidic conditions, the addition at the 6a-position was predominant, whereas the addition at the 6b-position was predominant under basic conditions. Over the pD range examined, the values of D_a_/D_b_ of 5*S*-DDdThd and 5*R*-DDdThd were almost identical. The median pD value for alternation of the D_a_/D_b_ value was approximately 8. Among the compounds in the present systems, acid-base equilibria with a p*K*_a_ value around 8 are as follows: For α-TP, the p*K*_a_ value between the monoanion and dianion on its phosphate group is reported as 6.7 [6]. For phosphoric acid, the p*K*_a_ value between dihydrogen phosphate H_2_PO_4_^−^ and hydrogen phosphate HPO_4_^2−^ is 7.2. For dThd, the p*K*_a_ value between the neutral molecule and monoanion on the thymine base moiety is 9.8 [26]. However, there is no information about acid-base equilibria of excited state or transient species of α-TP and dThd, which may exist in the UV-irradiated solution of the present study. It is not clear which acid-base equilibrium is responsible for alternation of the configuration of H additions to the 6 position of dThd.

It has been reported that although UV irradiation of dThd solution at 254 nm generates dThd cyclobutane dimers, it generates DHdThd as a major product in the presence of reducing agents such as NaHSO_3_ and NaH_2_PO_2_ [11, 27, 28]. In the presence of acetone, a photosensitizer, UV irradiation of dThd at 313 nm generates DHdThd and 5-acetonyl-DHdThd, an acetone adduct of dThd, as minor products in addition to dThd cyclobutane dimers [17]. Recently we have reported that in the presence of salicylic acid, a photosensitizer, and ascorbic acid, a reducing agent, DHdThd and two salicylic acid adducts of dThd are generated as major products with dThd cyclobutane dimers as minor products [16]. In the present study, UV irradiation at wavelengths longer than 300 nm of dThd in the presence of α-TP generated DHdThd. DHdThd was the major product, although the yield of DHdThd does not fully explain the consumption of dThd (Table 1), suggesting that dThd cyclobutane dimers and α-TP adducts of dThd may be generated as minor products. Combining the present results with previous studies, a possible reaction mechanism is as follows. Radicals are generated from α-TP and Trolox by UV irradiation. The radicals react with all the nucleosides with comparative efficiencies. In addition, α-TP, not Trolox, is excited by UV at wavelengths longer than 300 nm. The excited α-TP induces excitation of dThd *via* energy transfer. The excited dThd reacts with a ground state α-TP, a reducing agent, generating in DHdThd by addition of two hydrogen atoms with both syn and anti configurations. Although the ratio of syn and anti configurations alternated depending on pH of the solution, the mechanism for addition of two hydrogen atoms to dThd is unclear. Further studies are needed to clarify the total reaction mechanism generating DHdThd from dThd by UV irradiation in the presence of α-TP.

In conclusion, the present study showed that α-TP is a photosensitizer of nucleoside reactions by UV light at wavelengths longer than 300 nm. dThd is the most reactive among the examined nucleosides, and it generates DHdThd as the major product by the addition of two hydrogen atoms from water. The hydrogen atoms are added by both syn and anti configurations with the ratio depending on pH of the solution. Since α-TP exists in animal cells and body fluids, and is used as an ingredient of some cosmetics, we should pay attention to the genotoxicity of α-TP in terms of DNA damage caused by sunlight.

## Data Availability

Not applicable.

## Abbreviations

α-TP: α-Tocopherol phosphate
dCyd: 2′-deoxycytidine
dGuo: 2′-deoxyguanosine
dThd: thymidine
dAdo: 2′-deoxyadenosine
5*S*-DHdThd: (5*S*)-5,6-dihydrothymidine
5*R*-DHdThd: (5*R*)-5,6-dihydrothymidine
5*S*-DDdThd: (5*S*)-5,6-dideuteriothymidine
5*R*-DDdThd: (5*R*)-5,6-dideuteriothymidine
